# Helmet-Noninvasive Ventilation for Hospitalized Critically Ill COVID-19 Patients: Has Vaccination and the New Variants Changed Evidence?

**DOI:** 10.3390/nursrep12030051

**Published:** 2022-07-17

**Authors:** Hugo Neves, Vítor Parola, Rafael A. Bernardes, Joana Sousa, Adriana Coelho, Maria dos Anjos Dixe, Nuno Catela, Arménio Cruz

**Affiliations:** 1 The Health Sciences Research Unit: Nursing (UICISA: E), Nursing School of Coimbra, 3045-043 Coimbra, Portugal; hugoneves@esenfc.pt (H.N.); vitorparola@esenfc.pt (V.P.); adriananevescoelho@esenfc.pt (A.C.); acruz@esenfc.pt (A.C.); 2 Portugal Centre for Evidence-Based Practice: A Joanna Briggs Institute Centre of Excellence, 3045-043 Coimbra, Portugal; 3 Center for Innovative Care and Health Technology (ciTechCare), Polytechnic of Leiria, 2411-901 Leiria, Portugal; joana.sousa@ipleiria.pt (J.S.); maria.dixe@ipleiria.pt (M.d.A.D.); nuno.correia@ipleiria.pt (N.C.); 4 School of Health Sciences, Polytechnic of Leiria, 2411-901 Leiria, Portugal

**Keywords:** noninvasive ventilation, COVID-19, SARS-CoV-2, review

## Abstract

Noninvasive ventilation (NIV) is a technique for breathing support that significantly improves gas exchange and vital signs, reducing intubation and mortality rates. Helmets, unlike facemasks, allow for longer-term treatment and better ventilation, also being more cost-effective. As of today, we have found no reviews addressing this topic. This review aims to identify, map, and describe the characteristics of the use of noninvasive ventilation through helmet interface in critically ill COVID-19 adult patients hospitalized in acute care settings throughout the multiple moments that defined the COVID-19 pandemic. This scoping review will follow the methodology for scoping reviews proposed by JBI. A set of relevant electronic databases will be searched using terms such as COVID-19, helmet, and noninvasive ventilation. Two reviewers will independently perform the study selection regarding their eligibility. Data extraction will be accomplished using a researcher’s developed tool considering the review questions. Findings will be presented in tables and a narrative description that aligns with the review’s objective. This scoping review will consider any quantitative, qualitative, mixed-methods studies and systematic review designs for inclusion, focusing on the use of helmet on critically ill adult patients with COVID-19 hospitalized in acute care settings.

## 1. Introduction

Mechanical ventilation is the most-used supportive technique in intensive care units (ICU), being applied in critically ill patients who present, among others, an increased respiratory rate, increased work of breathing (WoB), asynchronous respiratory pattern, change in consciousness level, oxygen desaturation, hypercapnia, respiratory acidosis and circulation problems. It can either be invasive or noninvasive. Invasive mechanical ventilation involves the need for sedation, which can, for example, trigger hemodynamic risks or the development of nosocomial infections. As for noninvasive ventilation (NIV), it is considered the first choice of ventilator support in exacerbations of chronic obstructive pulmonary disease (COPD), enablement of weaning and extubation in patients with COPD, cardiogenic pulmonary oedema, and immunosuppressed patients [1]. Apart from the risks of invasive ventilation, NIV has demonstrated increased efficacy in decreasing patients’ dyspnea and effort during breathing, thus improving gas exchange and avoiding endotracheal intubation [2].

NIV is a breathing support technique that significantly improves gas exchange and vital signs, reducing intubation by 20% and mortality rates by 13% [3]. It is usually recommended for patients with COVID-19-related acute hypoxemic respiratory failure (AHRF) [4], which has the following main causes: hypoventilation, diffusion impairment, shunt, and ventilation-perfusion (V/Q) mismatch [5].

NIV has been used since the 1980s in patients with AHRF, with the first randomized studies on NIV in patients with community-acquired pneumonia being developed in 1999 [6,7]. This technique has become an important alternative and preventive measure to other ventilation mechanisms, such as endotracheal intubation in ICU contexts [8].

Despite the lack of efficiency in reducing WoB in hypoxemic respiratory failure, NIV has demonstrated efficiency when addressing WoB in mild to severe hypercapnic respiratory failure caused by COPD [9]. Furthermore, NIV and long-term home NIV (LTH-NIV) have been documented in managing COPD exacerbations and end-stage neuromuscular respiratory atrophy [10,11].

NIV can either be applied in adult patients [9] or in pediatric care [12] through six types of interfaces [13]: nasal mask, oro-nasal mask (or face masks), nasal pillow mask, oral mask, full face mask, and helmets.

Regardless of the widespread use of facemasks for NIV application, this interface can fail to guarantee good ventilation, thus leading to NIV failure [14]. This failure results from substantial oxygen leaks when the interface is not correctly adapted, leading to inadequate airway supply of the established O_2_ rate. Another issue associated with this type of interface relates to the discomfort associated with its application. Throughout the years, patient comfort with NIV interfaces has been continuously optimized in the presence of new devices.

According to some authors, helmets, unlike facemasks, in this sense, allow for longer-term treatment and better ventilation [15], also being more cost-effective [16]. This type of interface’s cost-effectiveness can reduce ICU admissions and length-of-stay compared to other interfaces [17,18]. Helmets have also proven to be cost-effective in ICU contexts. This interface can be used as an alternative to invasive ventilation during the weaning phase, specifically in reducing infection rates [19].

Some studies have already compared helmets to other interfaces [13], highlighting an increase in comfort, allowing free communication, easier drinking, and expectoration. Additionally, NIV with helmets seems to reduce by 33% the incidence of complications (e.g., skin lesions, air leakage, poor tolerance) related to NIV through other interfaces and improve relevant outcomes such as PaO_2_/FiO_2_ compared to patients with facemasks [20]. In pediatric care, continuous positive airway pressure (CPAP) with a helmet efficiently improves patients’ clinical condition with mild-to-moderate respiratory distress [12]. Nevertheless, in patients with acute respiratory failure (ARF), symptoms such as dyspnea were less prevalent in groups with facemasks [13].

The new coronavirus discovered in 2019 in the province of Wuhan (China) has rapidly developed into a global pandemic with high mortality rates due to acute respiratory illness. A study with 10,815 patients with respiratory complications found European mortality rates ranging from 13% to 72% [21].

As a highly contagious disease caused by the SARS-CoV-2 virus, COVID-19 manifests from a mild to severe respiratory illness [22]. With COVID-19 incidence rates skyrocketing, respiratory support resources have become scarce, leading to ethical issues related to allocating ventilators [23]. Since 5% of the COVID-19 patients had to be admitted to ICUs, it is estimated that around 71–90% need to be mechanically ventilated, leading to high demands and the development of alternative strategies [24]. With new COVID-19 waves still in perspective in some countries [25], specifically those with zero-COVID strategies such as China [26], NIV and helmet NIV may play an essential role in managing respiratory support for these patients, being the difference between life and death. Despite the concerns about aerosol dispersion, which led to early intubations with mechanical ventilation, and the discouraging of NIV modalities, such as helmets [24], more studies are starting to recommend using helmet NIV in COVID-19 patients [27]. Some studies have shown that NIV in COVID-19 acute critical care contexts (e.g., emergency departments, acute care wards, ICUs) can effectively avoid intubation in half of the infected patients [28], and reduce mortality rates, even in cases of acute hypoxemic respiratory failure [29,30].

Despite the benefits associated with a helmet NIV, as with other NIV interfaces, helmets require specific protocols, particularly regarding its application and weaning [17]. However, multiple studies present protocol options that reveal different algorithms, specific parameters, and set-ups [20].

The use of helmet NIV in critically ill COVID-19 patients with severe respiratory illness in acute care contexts is becoming more reported in the literature, with different protocols in all the stages of use (e.g., set-up, weaning) being documented [31]. Furthermore, we are witnessing a shift in how the COVID-19 case profiles present as a result of new variants and, more specifically, as a consequence of vaccination [32]. Thus, due to the dispersion of knowledge and to improve practice regarding helmet NIV in COVID-19 patients, this scoping review aims to map and acknowledge the specificities of the usage of this interface. Specifically, we aim to provide an understanding of the influence of the vaccination and the new variants, comparing the evidence reports according to a timeline that includes these milestones.

Mapping the use of helmet NIV, specifically what triggers(ed) the option for this interface, contexts where it is(was) used, how it is(was) set up and how weaning is(was) performed, the most common parameters, adverse events, and benefits reported, will help identify how knowledge shifted, the knowledge gaps that need to be addressed, future research priorities and trends, and what clinical guidelines need to be developed.

In this sense, we aim “to take a historical photograph” of how helmet-NIV usage regarding critically ill COVID-19 patients changed throughout the different moments that potentially changed the profile cases. Specifically, we aim to "photograph" how the new variants and vaccination changed helmet-NIV use.

This scoping review will follow the methodology projected by JBI to conduct scoping reviews and aims to examine and map the use of helmet NIV for ventilator support in critically ill COVID-19 patients with severe illness hospitalized in acute care settings according to a timeline that includes vaccination and the shift points between the most infectious and prominent variants of SARS-CoV-2. An initial search of MEDLINE (PubMed), CINAHL (EBSCO), the Cochrane Database of Systematic Reviews, the JBI Evidence Synthesis, PROSPERO, and Open Science Framework (OSF), has revealed that there are no conducted, current underway scoping, or systematic reviews that address this topic [33].

## 2. Review Question(s)

This scoping review aims to map the knowledge regarding the use of helmet NIV for ventilator support in critically ill adult patients with COVID-19 with severe illness according to the World Health Organization (WHO) clinical progression scale, hospitalized in acute care settings. More precisely, this scoping review seeks to understand how vaccination potentially influenced a shift in the evidence regarding helmet NIV and how knowledge changed between the most infectious and prominent variants of SARS-CoV-2 by answering the following questions:

1. What physiologic ventilation parameters are indicated to trigger helmet-NIV usage on critically ill COVID-19 patients with severe illness (e.g., SpO_2_ values)?
2. In what contexts of acute care is helmet NIV used for critically ill COVID-19 patients with severe illness (e.g., intensive care units, wards)?
3. What are the procedures reported for the helmet NIV set-up on critically ill COVID-19 patients with severe illness (e.g., measure neck circumference)?
4. What helmet-NIV ventilator set-ups are more commonly used in critically ill COVID-19 patients with severe illness (e.g., FiO_2_ levels, target PEEP + 30–50%).
5. What are the procedures reported for helmet-NIV weaning on critically ill COVID-19 patients with severe illness (e.g., PEEP increments)?
6. What adverse events/complications are identified in helmet-NIV usage on critically ill COVID-19 patients with severe illness (e.g., eye irritation)?
7. What benefits from helmet NIV are described by critically ill COVID-19 patients with severe illness (e.g., comfort)?

## 3. Inclusion Criteria

### 3.1. Participants

Patients with a measure of viral burden (quantitative PCR or cycle threshold) indicating the presence of SARS-CoV-2 and with a score of six or more according to the WHO clinical progression scale [22]. Specifically, this review will include studies focused on noninvasive ventilation through helmet interface as respiratory support for COVID-19 patients of all ages with acute severe respiratory illness.

### 3.2. Concept

Studies exploring noninvasive ventilation delivered by a helmet interface either as a solo or part of a combined treatment. A helmet interface can be defined as a helmet comprising a crystal-clear hood and a soft collar surrounding the patient’s head that can provide noninvasive ventilatory support, either as CPAP or noninvasive positive pressure [34]. Evidence illustrates helmets’ application in cardiogenic pulmonary oedema, pneumonia, COVID-19, post-extubation, and immune suppression [34]. Specifically, this review will include studies focused on when helmet NIV can be/was used, where it is/was used, the set-up reported when using this interface, the weaning, and the adverse events and benefits reported.

### 3.3. Context

Studies focused on acute care delivered in a clinical hospital environment. Specifically, we will consider studies focused on intensive care units, emergency departments, and COVID-19 wards.

### 3.4. Types of Sources

All types of studies will be considered, namely those with experimental and quasi-experimental designs. Analytical observational studies, including prospective and retrospective cohort studies, case-control studies, and analytical cross-sectional studies, will also be included. Descriptive observational designs, such as case series and individual case reports, will also be included.

Qualitative studies will also be considered, such as phenomenology, qualitative description, and action research.

Systematic reviews and clinical practice guidelines that meet the inclusion criteria will also be considered.

## 4. Methods

The review will comply with the methodology of JBI for scoping reviews [33]. The final review will be reported following the Preferred Reporting Items for Systematic Reviews guidelines and Meta-Analysis extension for Scoping Reviews (PRISMA-ScR) [35]. This protocol was registered in the Open Science Framework (OSF).

### 4.1. Search Strategy

The search strategy will locate published and unpublished primary studies, reviews, and guidelines.

The research team developed a search strategy and was then validated by an experienced reviewer outside the team, which followed the PRESS checklist [36]. According to JBI recommendations, a three-step search strategy will be applied [33]. 

Firstly, a pilot search was developed on MEDLINE (PubMed) and CINAHL Complete (EBSCOhost) to identify articles related to the research questions. Following this phase, keywords in titles and abstracts were used to complete the final search strategy for MEDLINE (PubMed) (Table 1), which will be adapted to each database’s thesaurus. Lastly, the references of the articles included in the review will be analyzed for potential new papers.

All languages will be considered. Although the authors only master Portuguese, English, and Spanish, other languages will be translated using an online translator (Google Translator and Linguee). If relevant for the review, the translated information will be included and flagged; otherwise, it will be excluded.

Studies published in 2019 will be included, considering the start of the COVID-19 pandemic.

The databases to be searched will include JBI EBP Database (OVID), MEDLINE (PubMed); CINAHL complete (EBSCOhost); Cochrane Central Register of Controlled Trials; Cochrane Database of Systematic Reviews; Scopus; Web of Science (WoS); LILACS. Studies from grey literature will be retrieved from Evidence-informed Policy Network (EVIPnet) from the WHO; Centers for Disease Control and Prevention (CDC); European Centre for Disease Prevention and Control (ECDC); National Institute for Health and Care Excellence (NICE).

### 4.2. Source of Evidence Selection

Records will be retrieved and stored in the Mendeley V1.19.8 (Mendeley Ltd., Elsevier, Netherlands), and duplicates removed. The review team will complete pilot testing before the title, abstract, and full-text screening. For stage one (title/abstract), 5% of the total search will be used to achieve approximately 75% agreement between reviewers. As for stage two (full-text), 2% of the full-text articles will be used to achieve the same amount of agreement. Two independent reviewers will screen the titles and abstracts to determine whether they meet the inclusion criteria. Potentially relevant papers will be fully retrieved, and citation details imported into the JBI System for the Unified Management, Assessment, and Review of Information (JBI SUMARI; JBI, Adelaide, Australia) [37]. Secondly, two independent reviewers will evaluate the full text of selected citations against the established criteria. An exclusion will be applied to full-text studies if inclusion criteria are not met. Reasons for exclusion will be described in the final report. Lastly, the references list of all the included studies in the review will be hand-searched. Any reviewer disagreements will be resolved at each stage through discussion or with a third reviewer. The author will be contacted in case of the inaccessible full article.

Findings will be reported in the final scoping review using the PRISMA-ScR flow diagram [35].

### 4.3. Data Extraction

Extracted data will be organized in a template developed according to JBI [33] and following the review questions. An extraction tool draft is shown in Table 2. The data extraction draft might be adjusted as needed during the review. As Levac and colleagues suggested [38], the consistency of data extraction depends on an a priori pilot charting of the first five to ten studies by independent reviewers. A third reviewer will resolve any disagreements.

Study authors will be contacted for further information in case of missing data. Because review studies will be included, in the situation of data duplication, reviewers will choose to report the primary study.

### 4.4. Data Analysis and Presentation

Data will be systematized in a tabular manner, depending on which is more suitable for this review’s objective. A descriptive summary will be provided in alignment with this scoping review’s aim [33].

Two comparative summaries will be provided in alignment with this scoping review’s objective [33]. One summary will address the before and after the COVID-19 vaccination started. A second summary will compare the evidence reported according to the most prominent variants of SARS-CoV-2.

We will present what is known, the literature gaps in the field, and the potential implications for health care and research. All authors will review the descriptive summary.

## Figures and Tables

**Table 1:** Search strategy for Medline (PubMed) conducted on 5 July 2022.

Search	Query	Record Retrieved
#1	Helmet [Title/Abstract]	4870
#2	((Noninvasive ventilation [Title/Abstract]) OR (NIV[Title/Abstract]) OR (non-invasive ventilation [Title/Abstract]) OR (Noninvasive Ventilation [MeSH Terms]) OR NIPPV[Title/Abstract])	10,091
#3	((COVID-19[MeSH Terms]) OR (SARS-CoV-2[MeSH Terms])) OR (((COVID-19[Title/Abstract]) OR (SARS-CoV-2[Title/Abstract])) OR (2019-nCoV [Title/Abstract]))	266,569
#4	((Helmet[Title/Abstract]) AND ((((Noninvasive ventilation[Title/Abstract]) OR (NIV[Title/Abstract])) OR (non-invasive ventilation[Title/Abstract]) OR CPAP[Title/Abstract]) OR (Noninvasive Ventilation[MeSH Terms] OR NIPPV[Title/Abstract]))) AND (((COVID-19[MeSH Terms]) OR (SARS-CoV-2[MeSH Terms])) OR (((COVID-19[Title/Abstract]) OR (SARS-CoV-2[Title/Abstract])) OR (2019-nCoV[Title/Abstract])))	69

**Table 2:** Data extraction tool.

**Scoping Review Details**	
Scoping review title Review objective(s) Review question(s)	
**Inclusion/Exclusion Criteria**	
Population	
Concept	
Types of evidence source	
**Evidence Source Details and Characteristics**	
Author(s)	
Year of publication	
Origin/country of origin (where the source was published or conducted)	
Aims/purpose	
Population and sample size	
**COVID-19 Timeline**	
Vaccination (e.g., before; after; before and after)	
Prominent SARS-CoV-2 variant (e.g., Omicron)	
**Details/Results Extracted from the Source of Evidence** (concerning the concept of the scoping review)	
Trigger parameter(s)	
Context	
Set up procedure(s)	
Ventilatory parameter(s)	
Weaning procedure(s)	
Adverse Events/Complications	
Benefits	
Observations (e.g., combined treatment, identified gaps)	

## Data Availability

No new data were created or analyzed in this study. Data sharing is not applicable to this article.
